# Ninjin’yoeito improves respiratory symptoms after lung cancer surgery: a prospective randomized study

**DOI:** 10.1007/s00595-024-02977-w

**Published:** 2024-12-24

**Authors:** Suguru Mitsui, Yugo Tanaka, Megumi Nishikubo, Takefumi Doi, Shinya Tane, Daisuke Hokka, Takumi Imai, Yoshimasa Maniwa

**Affiliations:** 1 Division of Thoracic Surgery, Kobe University Graduate School of Medicine, 7-5-2 Kusunoki-cho, Chuou-ku, Kobe, Hyogo 650-0017 Japan; 2 Clinical and Translational Research Center, Kobe University Hospital, Hyogo, Japan

**Keywords:** Kampo, Ninjin’yoeito, Lung cancer, Perioperative treatment

## Abstract

**Purpose:**

This study evaluated the efficacy of ninjin’yoeito for alleviating postoperative symptoms after lung cancer surgery.

**Methods:**

Overall, 140 patients who underwent lobectomy were randomized into a conventional treatment group and a ninjin’yoeito group. The primary endpoint was change in the Cancer Fatigue Scale (CFS) score from baseline and the secondary endpoints were the Cancer Dyspnea Scale (CDS) scores, the Kihon Checklist, and respiratory function.

**Results:**

The mean change in the CFS score 8 weeks postoperatively was − 5.56 in the ninjin’yoeito group and − 5.53 in the conventional treatment group (*P* = 0.425), but this outcome did not meet the primary endpoint. Changes in the mean CDS scores 8 weeks postoperatively were − 5.60 and − 3.38 in the ninjin’yoeito and conventional groups, respectively, with a difference of − 1.95 (*P* = 0.049). The changes in the mean vital capacity 8 weeks postoperatively were − 340.5 mL in the ninjin’yoeito group and − 473.5 mL in the conventional treatment group, with a difference of + 135.1 mL (*P* = 0.041). The ninjin’yoeito group had a significantly lower proportion of patients with malnutrition 16 weeks postoperatively than the conventional treatment group (*P* = 0.040).

**Conclusion:**

The results of this study show that ninjin’yoeito is effective for alleviating respiratory symptoms and improving malnutrition after lung cancer surgery.

## Introduction

Lung cancer is the leading cause of cancer-related mortality worldwide. The treatment for lung cancer includes surgery, chemotherapy with anticancer drugs, immunotherapy, and radiation therapy. The treatment method is selected based on a comprehensive review of the patient’s clinical characteristics such as histological type, cancer progression, and health status; however, surgical therapy is the mainstay of cancer management. Advances in medical technology have contributed to the postoperative recovery of patients undergoing primary lung cancer surgery, including minimally invasive thoracoscopic surgery [1–3]. However, patients still suffer respiratory issues such as decreased respiratory function and exercise tolerance [4]. Moreover, older patients, smokers, and others at high perioperative risk can undergo minimally invasive surgery with fewer complications, although they do require additional care [5, 6]. Some patients become frail and their quality of life is compromised after surgery. Patients with frailty are reported to be at a high risk of postoperative complications [7].

Respiratory rehabilitation can improve postoperative symptoms. This strategy can prevent the development of respiratory complications and promote the recovery of physical activity in the early postoperative period, aiming to promote the reacquisition of activities of daily living. Preoperative rehabilitation can stabilize exercise tolerance and general condition in patients with primary lung cancer, who are scheduled to undergo lung resection [8, 9]. However, even with rehabilitation, a decline in postoperative status cannot be prevented. A randomized controlled trial of post-thoracotomy pulmonary rehabilitation for patients with resectable lung cancer revealed that their exercise tolerance improved, but their quality of life did not [10]. Moreover, the direct effect of rehabilitation on frailty is unclear and there are limited studies on pharmacotherapy. Ninjin’yoeito is a Japanese herbal medicine comprising 12 herbal ingredients, which improves appetite and physical weakness, and can be effective against postoperative illness, weakness, fatigue, anorexia, sweating, cold hands and feet, and anemia [11]. Previous reports have shown that ninjin’yoeito is also useful for improving respiratory symptoms [12]. A prospective study revealed that ninjin’yoeito is effective for treating general weakness and physical symptoms such as fatigue and anorexia in elderly individuals [13]. Thus, we conducted an indiscriminate comparative study to validate the usefulness of ninjin’yoeito for preventing postoperative frailty after lung cancer surgery.

## Methods

### Study design

This single-center, open-label, randomized, controlled trial evaluated the efficacy of ninjin’yoeito against perioperative physical symptoms in patients with primary lung cancer, who underwent lobectomy and lymph node dissection between September, 2020 and February, 2024. The protocol was established in accordance with the principles of the Declaration of Helsinki. The current study was approved by the Clinical Research Area Ethics Committee of Kobe University Graduate School of Medicine (#C190015) and was registered in the Japan registry of clinical trials (jRCTs051200009).

### Study population

This study included patients aged > 20 years who were scheduled to undergo lobectomy for suspected clinical stage I primary lung cancer under VATS (video-assisted thoracic surgery) or RATS (robot-assisted thoracic surgery). The primary exclusion criteria were as follows: patients with a history of allergy to ninjin’yoeito or its ingredients, those who had taken herbal medicine preparations within 2 weeks, those with low hepatic or renal function, and those not eligible for the study based on the investigators’ discretion. The postoperative exclusion criteria were as follows: patients who underwent thoracotomy, those who underwent resection of greater than two lobes, and those who could not start drinking fluids within 3 days after surgery. Patients who did not meet the postoperative exclusion criteria were randomized. Patient information was extracted through physical examination, medical history taking, respiratory function testing, chest radiography examination, and peripheral blood analysis. All preoperative evaluations were performed routinely within 56 days before surgery.

### Ninjin’yoeito

Ninjin’yoeito Extract Granules Kracie® (Kracie Pharmaceutical Ltd., Japan) were used in this study. Ninjin’yoeito comprises 12 herbal medicines including ginseng, Japanese angelica root, peony root, rehmannia root, atractylodes rhizome, poria sclerotium, cinnamon bark, astragalus root, citrus unshiu peel, polygala root, schisandra fruit, and glycyrrhiza. Participants who had one of the indicators for ninjin’yoeito (physical strength decline, fatigue, anorexia, perspiration during sleep, cold limbs, and anemia) received the medicine.

### Procedure

The participants were assigned randomly to either the conventional treatment group or the conventional treatment plus ninjin’yoeito group (ninjin’yoeito group) at an allocation ratio of 1:1. The ninjin’yoeito group patients started taking ninjin’yoeito (7.5 g/day) in two divided doses, before breakfast and before dinner, for 16 weeks after resuming oral fluids postoperatively. Peripheral blood analysis, questionnaire survey, and weight measurements were taken after resuming oral fluid intake at the start of internal administration and then 1, 8, and 16 weeks postoperatively. Respiratory function tests were performed 8 weeks postoperatively. Peripheral blood analysis, a questionnaire survey, and weight measurements at the start of internal administration were defined as the baseline parameters. The changes in each item from the baseline were assessed 1, 8, and 16 weeks postoperatively.

### Outcomes

The primary evaluation visit was 8 weeks postoperatively and the primary endpoint was the change in the total Cancer Fatigue Scale (CFS) score from the baseline. The secondary endpoints included dyspnea measured using the Visual Analog Scale (VAS), the Cancer Dyspnea Scale (CDS) score, the MD Anderson Symptom Inventory (MDASI) score, the Kihon Checklist (KC) score, respiratory function test findings, weight measurements, and peripheral blood analysis results.

The CFS is a 15-item scale comprising 3 subscales (physical, emotional, and cognitive) used to assess fatigue in cancer patients. [14]. Higher CFS scores indicate stronger fatigue. The VAS can quantify the assessment of pain using method that plots the intensity of pain on a straight line [15]. The CDS score can be used to assess three subjective measures: respiratory effort, anxiety, and discomfort [16]. A higher CDS score indicates worse dyspnea. The MDASI is a 19-item questionnaire used to assess patients with cancer [17]. This scoring system has 13 items for evaluating the intensity of symptoms and the disruption in activities of daily living. The KC is a comprehensive self-report health checklist designed by the Japanese government’s Ministry of Health, Labour and Welfare, and used as a predictive tool for frailty [18].

### Monitoring

Monitoring was done to assess whether the study was conducted in accordance with the protocol, and the data were collected appropriately. An assigned individual who was not a researcher in the current study reviewed the consent forms from the participants, the medical records, and the case report forms of all participants.

### Randomization

Randomization was performed using a randomized table generated independently from the researchers, using permuted block randomization with a block size mixture of 4 and 6.

### Statistical analysis

There have been no previous clinical studies published on ninjin’yoeito. Thus, a previous study that evaluated fatigue in patients with advanced-stage cancer using the CFS after intervention with reflexology was used as a reference [19]. The intervention methods differ, but if the efficacy of ninjin’yoeito is not similar to that of reflexology, its significance could be limited. The mean and standard deviation of the difference in the CFS scores before and after the intervention was 4.6 ± 9.6. Using this value as a reference, the mean CFS score between the intervention and control groups was found to be 4.6, with a significance level of 5% and a power of 80%. In total, 69 patients were required per group. Considering the number of cases that can be collected in our department, the sample size was set at 70 patients per group, with a total of 140, to achieve a power of 80%.

The CFS was correlated with the 6-min walking distance (6MWD), with a two-point improvement in CFS corresponding to a 58-m increase in 6MWD [20]. Furthermore, an improvement of > 50 m in the 6MWD is necessary for patients to perceive an improvement in their exercise capacity. Therefore, if the administration of ninjin’yoeito results in a 4.6-point improvement in CFS, it could lead to a recovery in walking ability, perceived improvement in exercise capacity, increased activity levels, and a smoother return to society.

All analyses were performed based on the intention-to-treat principle. The demographic characteristics of the patients are expressed as means and the standard deviation for continuous variables and as the frequency and proportion for categorical variables. All continuous outcomes were analyzed using the mixed-effects models for repeated measurements with a compound symmetry covariance structure. The presence or absence of issues in the KC was analyzed using a generalized linear model with a binomial distribution and an identity link function, using robust standard errors. All statistical models included the indicators of the group and visit, their interaction, and baseline values as covariates. All statistical tests were evaluated at a two-sided significance level of 0.05. Analyses were performed using the R software version 4.1.0 (R Foundation for Statistical Computing, Vienna, Austria) and the SAS software version 9.4 (SAS Institute, Cary, NC, the USA).

## Results

**Fig. 1:**
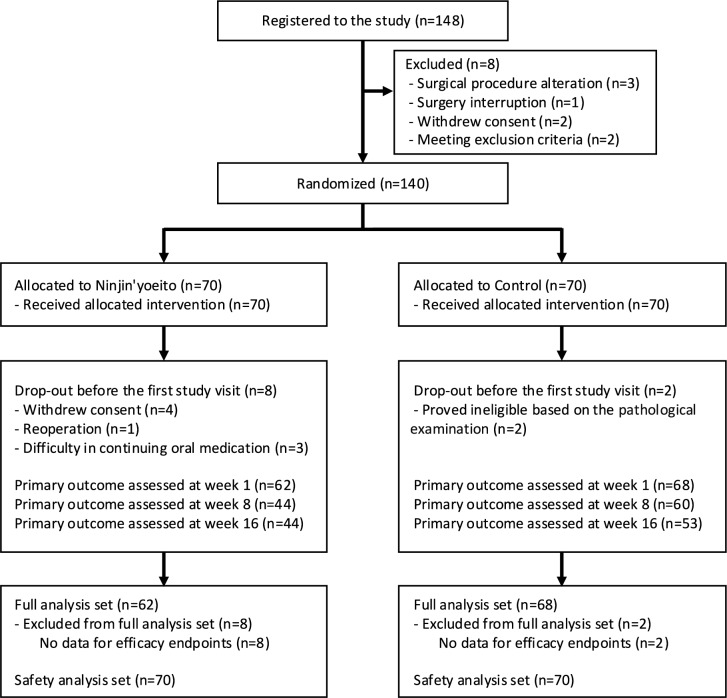
Flowchart of the inclusion of patients in this study

Figure 1 shows the flowchart of the inclusion of patients in this study. Of the 148 patients who met the primary enrollment criteria, 140 who met the inclusion criteria were enrolled and randomized into the ninjin’yoeito (*n* = 70) and conventional treatment (*n* = 70) groups. After excluding patients who left before the first study visit, 130 (62 in the ninjin’yoeito group and 68 in the conventional treatment group) were included in the analysis set. At 8 weeks, 44 patients in the ninjin’yoeito group and 60 in the conventional treatment group were assessed for the primary endpoint.

Table 1 summarizes the characteristics of the patients. There were 70 male and 60 female participants, with a mean age of 69 years. The mean weight and body mass index (BMI) of the patients were 60.2 kg and 23.1 kg/m^2^, respectively. The numbers of patients undergoing video-assisted thoracic surgery and robot-assisted thoracic surgery were 49 (37.7%) and 81 (62.3%), respectively. There were 110 (84.6%) patients with adenocarcinoma, 10 (7.7%) with squamous cell carcinoma, and 10 (7.7%) with other types of carcinoma.

**Table 1:** Clinical characteristics of the patients

Characteristics	All (*N* = 130)	Ninjin’yoeito group (*N* = 62)	Conventional group (*N* = 68)
	Mean ± SD or N (%)	Mean ± SD or *N* (%)	Mean ± SD or *N* (%)
Age (years)	69.1 ± 9.4	69.3 ± 10.3	68.9 ± 8.4
Sex			
Male	70 (53.8)	34 (54.8)	36 (52.9)
Female	60 (46.2)	28 (45.2)	32 (47.1)
Body weight (kg)	60.2 ± 12.7	59.4 ± 10.7	60.9 ± 14.3
Body mass index (kg/m^2^)	23.1 ± 4.5	22.8 ± 3.5	23.3 ± 5.2
Preoperative pulmonary function			
VC (mL)	3217 ± 842	3215 ± 809	3219 ± 877
FEV1.0 (mL)	3150 ± 836	3159 ± 805	3142 ± 869
FEV1.0% (%)	74.9 ± 8.6	76.6 ± 7.2	73.4 ± 9.4
DLCO% (%)	88.1 ± 22.4	90.4 ± 20.9	86.0 ± 23.7
Tumor location			
Right upper	51 (39.2)	22 (35.5)	29 (42.6)
Right middle	16 (12.3)	8 (12.9)	8 (11.8)
Right lower	18 (13.8)	8 (12.9)	10 (14.7)
Left upper	22 (16.9)	12 (19.4)	10 (14.7)
Left lower	23 (17.7)	12 (19.4)	11 (16.2)
Surgical procedure			
VATS	49 (37.7)	21 (33.9)	28 (41.2)
RATS	81 (62.3)	41 (66.1)	40 (58.8)
Tumor size (mm)^a^	22.7 ± 9.0	22.6 ± 7.9	22.8 ± 10.0
Histology^a^			
Adenocarcinoma	110 (89.4)	52 (89.7)	58 (89.2)
Squamous cell carcinoma	10 (8.1)	5 (8.6)	5 (7.7)
Others	3 (2.4)	1 (1.7)	2 (3.1)
Pathological stage			
0	7 (5.4)	2 (3.2)	5 (7.4)
IA	80 (61.5)	38 (61.3)	42 (61.8)
IB	19 (14.6)	11 (17.7)	8 (11.8)
IIA	4 (3.1)	2 (3.2)	2 (2.9)
IIB	9 (6.9)	4 (6.5)	5 (7.4)
IIIA	3 (2.3)	1 (1.6)	2 (2.9)
IIIB	1 (0.8)	0	1 (1.5)
Others	7 (5.4))	4 (6.5)	3 (4.4)
Major adverse events			
Interstitial pneumonia	1 (0.8)	1 (1.6)	0
Minor adverse events			
Prolonged air leakage	5 (3.8)	3 (4.8)	2 (2.9)
Atrial fibrillation	4 (3.1)	2 (3.2)	2 (2.9)
Wound infection	2 (1.5)	0	2 (3.2)

^a^Data were missing for seven patients (*n* = 4 from the ninjin'yoeito group, *n* = 3 from the conventional group)

SD, standard deviation; VC, vital capacity; FEV1.0, forced expiratory volume in one second; FEV1.0%, forced expiratory volume % in 1 s; DLCO, single-breath diffusing capacity of the lung for carbon monoxide; VATS, video-assisted thoracic surgery; RATS, robot-assisted thoracic surgery

**Fig. 2:**
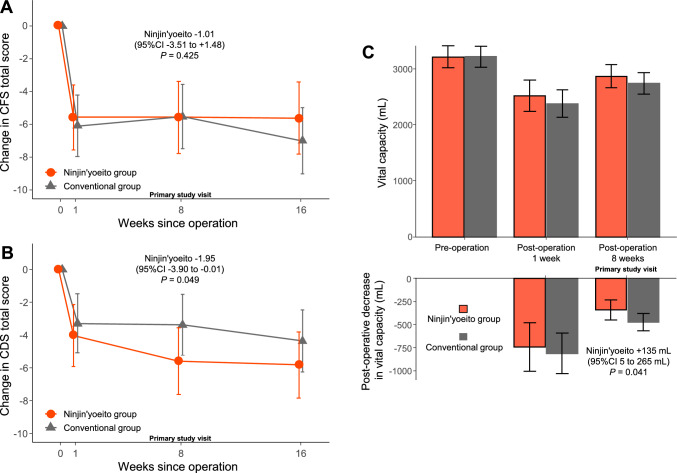
Changes in the total Cancer Fatigue Scale (CFS) (**a**) and Cancer Dyspnea Scale (CDS) (**b**) scores. The change in the total CFS score 8 weeks postoperatively did not differ between the two groups (**a**). The ninjin’yoeito group had a significantly better total CDS score at 8 weeks than the conventional treatment group (**b**). The vital capacity and postoperative decrease in the vital capacity (**c**). The ninjin’yoeito group had a significantly greater postoperative decrease in vital capacity at 8 weeks than the conventional treatment group

**Fig. 3:**
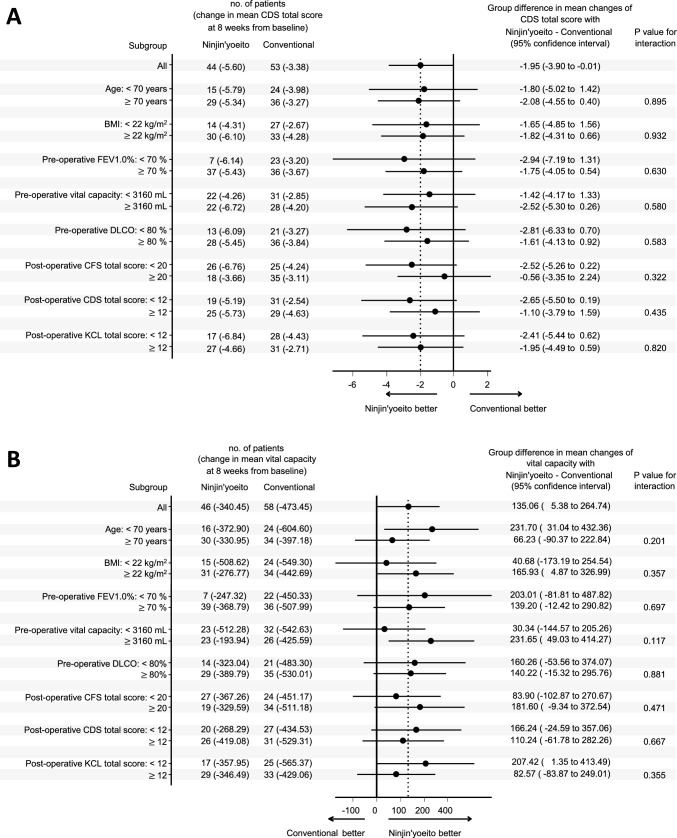
Subgroup analysis of the changes in the total Cancer Dyspnea Scale (CDS) score (**a**) and vital capacity (**b**). There were no significant differences in terms of the *P* value for interaction

The ninjin’yoeito and conventional treatment groups had a better CFS score postoperatively (Fig. 2A). The changes in the mean CFS scores at 8 weeks were − 5.56 in the ninjin’yoeito group and − 5.53 in the conventional treatment group, with a difference of − 1.01 between the groups [95% confidence interval (CI) − 3.51 to 1.48, *P* = 0.425]. There was similar improvement in the CDS score postoperatively, but it was better in the ninjin’yoeito group (Fig. 2B). The changes in the mean CDS scores at 8 weeks were − 5.60 in the ninjin’yoeito group and − 3.38 in the conventional treatment group, with a difference of − 1.95 between the groups (95% CI − 3.90 to − 0.01, *P* = 0.049). In terms of respiratory function, the ninjin’yoeito group had greater recovery of vital capacity at 8 weeks than the conventional treatment group (Fig. 2C). The postoperative changes in the mean vital capacity at 8 weeks were − 340.5 mL in the ninjin’yoeito group and − 473.5 in the conventional treatment group, with a difference of + 135.1 mL between the groups (95% CI 5.4–264.7 mL, *P* = 0.041). There were no significant differences in terms of the total KC score, other respiratory function parameters, or body weight (Table 2). Moreover, the two groups did not differ significantly in terms of dyspnea measured using the VAS, MDASI, and peripheral blood analysis. Figure 3 shows the results of the post hoc subgroup analysis for CDS and vital capacity, where the effect of ninjin’yoeito was observed. Based on these results, there was no significant heterogeneity in the effect of ninjin’yoeito.

**Table 2:** Baseline values and the change 8 weeks postoperatively

Outcomes	Visit	Ninjin’yoeito group (*N* = 62)		Conventional group (*N* = 68)		Between-group difference	
		*n*	Estimate (95%CI)		Estimate (95%CI)	Estimate (95%CI)	*P* value
CFS total score	Mean at baseline	62	19.7 (17.6 to 21.8)	68	21.8 (19.8 to 23.8)		
	Change at 8 weeks	44	− 5.6 (− 7.8 to − 3.4)	60	− 5.5 (− 7.5 to − 3.6)	− 1.0 (− 3.5 to 1.5)	0.425
CDS							
Effort	Mean at baseline	62	5.4 (4.5 to 6.3)	68	5.2 (4.3 to 6.0)		
	Change at 8 weeks	44	− 2.2 (− 3.3 to − 1.2)	60	− 1.1 (− 2.0 to − 0.1)	− 1.0 (− 2.0 to 0.1)	0.07
Anxiety	Mean at baseline	62	5.7 (5.0 to 6.3)	68	6.0 (5.4 to 6.6)		
	Change at 8 weeks	44	− 2.0 (− 2.8 to − 1.1)	60	− 1.5 (− 2.3 to − 0.7)	− 0.6 (− − 1.5 to 0.3)	0.161
Discomfort	Mean at baseline	62	1.9 (1.4 to 1.6)	68	1.6 (1.1 to 2.1)		
	Change at 8 weeks	44	− 1.5 (− 2.1 to − 0.8)	60	− 0.8 (− 1.4 to − 0.2)	− 0.4 (− 0.9 to 0.2)	0.223
Total score	Mean at baseline	62	12.9 (11.3 to 14.6)	68	12.8 (11.2 to 14.4)		
	Change at 8 weeks	44	− 5.6 (− 7.6 to − 3.6)	60	− 3.4 (− 5.2 to − 1.5)	− 2.0 (− 3.9 to − 0.0)	0.049
Kihon Checklist total score	Mean at baseline	62	12.8 (12.0 to 13.6)	67	12.3 (11.6 to 13.1)		
	Change at 8 weeks	44	− 0.7 (− 1.5 to 0.2)	57	0.1 (− 0.7 to 0.8)	− 0.5 (− 1.6 to 0.5)	0.294
Preoperative pulmonary function							
VC (mL)	Mean at baseline	62	3214.7 (3019.2 to 3410.1)	67	3218.8 (3030.8 to 3406.8)		
	Change at 8 weeks	46	− 340.5 (− 447.8 to − 233.1)	58	− 473.5 (− 568.8 to − 378.1)	135.1 (5.4 to 264.7)	0.041
FEV1.0 (mL)	Mean at baseline	62	2410.0 (2265.4 to 2554.6)	67	2274.0 (2134.9 to 2413.2)		
	Change at 8 weeks	46	− 286.2 (− 383.7 to − 188.8)	58	− 337.4 (− 424.0 to − 250.7)	83.7 (− 12.3 to 179.7)	0.087
FEV1.0% (%)	Mean at baseline	62	76.6 (74.4 to 78.7)	67	73.4 (71.3 to 75.4)		
	Change at 8 weeks	46	− 1.1 (− 3.2 to 1.1)	58	− 1.1 (− 3.0 to 0.8)	0.9 (− 1.7 to 3.5)	0.484
DLCO% (%)	Mean at baseline	62	90.3 (85.0 to 95.6)	67	85.8 (80.8 to 90.9)		
	Change at 8 weeks	46	− 11.1 (− 16.6 to − 5.7)	58	− 13.0 (− 17.8 to − 8.2)	3.4 (− 2.0 to 8.8)	0.218
Body weight (kg)	Mean at baseline	62	59.4 (56.2 to 62.6)	67	60.9 (57.9 to 64.0)		
	Change at 8 weeks	44	− 1.7 (− 2.4 to − 1.0)	57	− 1.6 (− 2.3 to − 1.0)	− 0.1 (− 1.1 to 0.9)	0.797

CI, confidence interval; CFS, Cancer Fatigue Scale; CDS, Cancer Dyspnea Scale; VC, vital capacity; FEV1.0, forced expiratory volume in 1 s; FEV1.0%, forced expiratory volume % in 1 s; DLCO, single-breath diffusing capacity of the lung for carbon monoxide

Table 3 shows the results of the KC at baseline, and 8 and 16 weeks postoperatively in the ninjin’yoeito and conventional treatment groups. By 16 weeks postoperatively, the ninjin’yoeito group had a significantly lower proportion of patients with malnutrition (nutrition status based on the KC) than the conventional treatment group. The other parameters did not differ significantly between the groups.

**Table 3:** Baseline values and results of the Kihon Checklist 8 and 16 weeks postoperatively

Outcome	Visit	Ninjin'yoeito group (*n* = 62)		Conventional group (*n* = 68)		*P* value
		*n*	Patients with problem *n* (%)	*n*	Patients with problem *n* (%)	
Daily activities	Baseline	62	20 (32.3)	68	14 (20.6)	
	8 weeks	44	7 (15.9)	60	13 (21.7)	0.187
	16 weeks	44	14 (31.8)	53	12 (22.6)	0.416
Physical function	Baseline	62	53 (85.5)	68	45 (66.2)	
	8 weeks	44	37 (84.1)	60	45 (75.0)	0.662
	16 weeks	44	39 (88.6)	53	40 (75.5)	0.358
Nutrition	Baseline	62	2 (3.2)	67	3 (4.5)	
	8 weeks	44	3 (6.8)	57	5 (8.8)	0.670
	16 weeks	44	0 (0.0)	52	5 (9.6)	0.040
Oral function	Baseline	62	13 (21.0)	68	21 (30.9)	
	8 weeks	44	10 (22.7)	60	13 (21.7)	0.402
	16 weeks	44	12 (27.3)	53	13 (24.5)	0.137
Socialization	Baseline	62	7 (11.3)	68	9 (13.2)	
	8 weeks	44	3 (6.8)	60	9 (15.0)	0.078
	16 weeks	44	2 (4.5)	53	7 (13.2)	0.362
Cognition	Baseline	62	61 (98.4)	68	62 (91.2)	
	8 weeks	44	41 (93.2)	60	57 (95.0)	0.614
	16 weeks	44	42 (95.5)	53	52 (98.1)	0.336
Depression	Baseline	62	25 (40.3)	68	35 (51.5)	
	8 weeks	44	11 (25.0)	60	20 (33.3)	0.743
	16 weeks	44	8 (18.2)	53	14 (26.4)	0.466

Regarding major adverse events, one patient had an illness not attributed to this clinical study. During the postoperative outpatient follow-up, the patient was found to have interstitial shadows in the lung and was hospitalized for drug-induced interstitial pneumonia caused by adjuvant chemotherapy with uracil–tegafur, diagnosed through allergy testing. The patient recovered within a few days and was discharged from hospital. Minor adverse events included prolonged air leakage in five patients, atrial fibrillation in four patients, and wound infection in two patients, none of which were attributable to the study and all resolved promptly.

## Discussion

The perioperative symptoms of lung cancer include pain, dyspnea, cough, fatigue, depression, anxiety, and malnutrition, and their protraction poses a high burden on postoperative quality of life [21, 22]. Thus, attempts have been made to optimize the quality of life of patients with lung cancer in the perioperative period. Previous studies have revealed improvements in long-term quality of life through pain control, and in postoperative pulmonary and physical function through exercise intervention [23, 24]. Nutritional therapy and rehabilitation are also being used to treat frailty [25–27]. However, the treatment options for the postoperative symptoms of lung cancer are limited and there are only a few published studies on pharmacological treatment. This prospective randomized trial aimed to evaluate the efficacy of ninjin’yoeito for alleviating perioperative physical symptoms in patients undergoing lobectomy for primary lung cancer. Prospective randomized studies comparing the clinical application of Kampo for perioperative symptoms are limited but worthwhile.

The total CFS score, which was the primary endpoint of the study, did not differ significantly between the two groups. The CFS score, which quantifies frailty based on function, is often used as an endpoint in different populations that represent the elderly [28]. In this study, the CFS score was used as the primary endpoint because a previous retrospective study on ninjin’yoeito showed a difference in the CFS score [12]. The CFS score is a combination of clinical judgment and objective measurement and is evaluated based on various items. Hence, differences were less likely if the total score was calculated even in patients who were frail in some aspects. Moreover, the variation in the CFS score at baseline between the two groups after randomization might have contributed to the lack of difference in this study.

Regarding the efficacy of ninjin’yoeito for increasing appetite and improving a weak constitution, previous research has shown evidence of improvement in anorexia scores in patients with Alzheimer’s disease and in fatigue [29], malaise, and anorexia in people aged > 65 years [13]. The mechanisms of improvement in anorexia with ninjin’yoeito include activation of the ghrelin-responsive and ghrelin-unresponsive neuropeptide Y pathways and orexigenic orexin 1 receptor-expressing neurons in the hypothalamus, and the promotion of increasing intracellular dopamine content by inhibiting metabolic enzymes [30–32]. In this study, there were no significant differences in body weight or albumin levels in patients who received ninjin’yoeito; however, the proportion of patients with malnutrition based on the KC decreased significantly. Malnutrition in the KC is evaluated in two ways based on whether the BMI is < 18.5 kg/m^2^ or higher and whether there is weight loss of > 2–3 kg in 6 months [18]. In the current study, 39 patients had a BMI of ≤ 22, and only 5 patients had malnutrition based on the KC at baseline. The limited number of patients with poor nutritional status might have contributed to the lack of differences in albumin levels and body weight. However, the decrease in the proportion of patients with malnourishment may best reflect the effect of ninjin’yoeito. Moreover, the evaluation within the 16-week period may have been insufficient to assess changes in weight and albumin levels, partly because of the perioperative modifications. Previous studies showing evidence of improvements in weight gain and albumin levels because of ninjin’yoeito are limited.

This study included only patients undergoing lobectomy, which is the standard surgical procedure for lung cancer; therefore, the proportion of patients with frailty was small. However, patients with frailty are difficult to enroll, and even the physical fitness of healthy individuals is compromised by lobectomy. Therefore, we consider there is potential for intervention to promote early reintegration into society and reduce complications. Given that the effects of perioperative drug intervention for recovery from fatigue have not yet been investigated, we evaluated whether ninjin’yoeito would be effective in patients who undergo pulmonary resection. We selected lobectomy, which is not too invasive, because the effectiveness of postoperative interventions is more likely to be observed after this procedure. The favorable results of this study, which included a large proportion of patients without frailty, are significant. Our patients’ CDS score and vital capacity improved with ninjin’yoeito. CDS has been used for evaluating patients with cancer in several studies, but the minimum clinically significant difference in CDS scores has not been established [33]. Although the difference in CDS scores was only 1.95 points out of a possible score of 48 points, previous studies have shown that a difference of 2–3 points in CDS scores is significant [34, 35]. Furthermore, considering that the mean baseline score for CDS is 12 points, a difference of 1.95 points is not small; therefore, 1.95 points may be sufficiently significant in this study. Previous studies on the improvement of respiratory symptoms by ninjin’yoeito include a retrospective analysis of fatigue caused by dyspnea in patients with interstitial pneumonia. The results of these studies showed that ninjin’yoeito could improve the CFS score significantly; however, multivariate analysis did not validate any factor that influenced the changes in the CFS score [12]. The use of ninjin’yoeito in a COPD mouse model suppressed the loss of muscle mass and alteration in the muscle fiber distribution by increasing expression of the peroxisome proliferator-activated receptor gamma coactivator-1 alpha, which is involved in skeletal muscle function [36]. A similar study found that the schisandra fruit in ninjin’yoeito improved endurance and energy metabolism in exercise rats and increased expression of the peroxisome proliferator-activated receptor gamma coactivator-1, a regulator of energy metabolism in skeletal muscle [37]. The difference in vital capacity and even CDS between the two groups was attributed to the inhibition of muscle loss and alteration in the muscle fiber distribution by the above mechanisms, which enabled stability of the respiratory muscles. Based on the surgical procedure, respiratory function and exercise tolerance after lung surgery were more likely to decrease for 3–6 months postoperatively, and then improve [38, 39]. Ninjin’yoeito may contribute to the early improvement of postoperative respiratory symptoms in the postoperative period and even within 16 weeks, thereby preventing acute postoperative pulmonary complications and contributing to the reduction of societal costs.

Kampo’s adverse events include interstitial pneumonia and liver dysfunction, allergic cystitis, and drug eruption. Interstitial pneumonia and liver dysfunction, which are serious side effects, are caused by an immune allergic reaction, the main cause of which could be formulas containing Scutellariae Radix [40]. Scutellariae Radix is not included in ninjin’yoeito and we observed no serious adverse events attributable to ninjin’yoeito; however, patients with a history of side effects such as interstitial pneumonia and liver damage from Kampo are at a high risk of Kampo’s adverse events and require special attention.

The present study had some limitations. First, it was conducted at a single center, so the generalizability of the results was not validated. Second, the trial was conducted in randomized settings. Initially, a blinded method was planned but was deemed impractical because of the number of capsules that had to be taken when making placebo drugs in Kampo. Although the placebo effects of the taste characteristics of ninjin’yoeito cannot be ruled out because of the study’s open-label nature, the fact that various items, including the KC, MDASI, and VAS, revealed no significant differences and that differences were found in objective measures, including the respiratory function test that we conducted, suggests that the placebo effect was limited. There were some baseline differences including the primary endpoints of CFS and respiratory test findings between the two groups. There are several possible reasons for this, including the small sample size and dropouts after randomization. To address this issue, baseline values were adjusted in the analyses to calculate the estimates. Furthermore, several patients left the study within 8 weeks for reasons such as withdrawing consent after enrollment and difficulty taking the medication because it was unpalatable. This can be explained by the fact that Kampo has a peculiar smell and taste, making it difficult to tolerate. Although adequate statistical power was ensured, a more accurate assessment would have been possible if the follow-up of these patients had been better. Of note, during the planning stage of the trial, considerations regarding the clinically significant minimum effect size were conducted only for the primary outcomes and not for the secondary outcomes. The third limitation pertains to the evaluation time and timing of testing. The evaluation time was initially planned to be 24 weeks, but recurrence might transpire within 24 weeks postoperatively, in which case, fatigue and malaise might be triggered by simply informing the patient about recurrence. Thus, the results recorded 24 weeks postoperatively might be inconsistent with the results of the primary endpoint at 8 weeks and therefore, we decided on the observation period of 16 weeks. Moreover, we originally planned to examine respiratory function 1, 8, and 16 weeks postoperatively. Because of the COVID-19 pandemic and the limited number of respiratory function tests being performed for the infection control measures in the hospital, postoperative respiratory function tests could not be carried out as prescribed. Fourth, this study included only patients who underwent lobectomy with more than a certain level of invasiveness to assess for physical, mental, and social functional decline caused by surgical invasiveness. Given that the resection volume of the middle lobe was smaller than that of the other lobes, the postoperative functional decline may have been smaller than that in the other lung lobes, and the difference between the two groups might have been greater if the middle lobectomy had been excluded.

In conclusion, ninjin’yoeito did not improve fatigue; however, it could be effective in alleviating respiratory symptoms and malnutrition after lung cancer surgery. Major adverse events were not observed in the perioperative period in this study. Therefore, ninjin’yoeito can reduce the risk of acute postoperative complications and decrease social costs for patients at high risk of postoperative complications after lung cancer surgery.
